# The role of serum vitamin D in patients with normal ovarian reserve undergoing the first IVF/ICSI cycle

**DOI:** 10.3389/fendo.2023.1249445

**Published:** 2023-08-24

**Authors:** Rong Luo, Jiahui Wang, Yu Yang, Cen Xu, Minyan Yang, Dandan Zhu, Jia Wang, Ping Zhang, Hongshan Ge

**Affiliations:** ^1^ Department of Reproductive Medicine, The Affiliated Taizhou People’s Hospital to Nanjing Medical University, Taizhou, China; ^2^ School of Medicine, Southeast University, Nanjing, China

**Keywords:** vitamin D, IVF, pregnancy, embryo transfer, assisted reproduction

## Abstract

**Background:**

The debate over the impact of vitamin D in assisted reproduction continues. The purpose of our study was to assess embryo quality and pregnancy outcomes among groups with different levels of vitamin D after the first *in vitro* fertilization (IVF)/intracytoplasmic sperm injection (ICSI) cycle in patients with normal ovarian reserve (NOR).

**Methods:**

Patients in this retrospective cohort study were divided into three groups: severe vitamin D deficiency group (25OH-D < 10 ng/ml), vitamin D deficiency group (10 ng/ml ≤ 25OH-D < 20 ng/ml), and non-vitamin D deficiency group (25OH-D ≥ 20 ng/ml). The primary outcome was clinical pregnancy, while the secondary outcomes were mature oocytes, oocyte fertilization, available cleavage embryos, available blastocysts, biochemical pregnancy, early abortion, and embryo implantation. A modified Poisson regression model and multiple linear regression analysis were conducted for the multivariate analysis.

**Results:**

264 NOR patients undergoing the first IVF/ICSI cycles were included. For the primary outcome, there was no significant difference in clinical pregnancy between the severe vitamin D deficiency group and the other two groups (vitamin D deficiency group: adjusted RR = 1.026; 0.780 - 1.350; P = 0.854; non-vitamin D deficiency group: adjusted RR = 1.092; 0.743 - 1.605; *P* = 0.652). For all secondary outcomes, no significant differences were observed among the severe vitamin D deficiency, vitamin D deficiency, and non-vitamin D deficiency groups (*P* > 0.05). Exploratory subgroup analyses concerning the season of embryo transfer, phase of embryo transferred, and endometrial thickness, as well as the sensitivity analysis using logistic regression models for the primary outcome, revealed comparable clinical pregnancy rates among the groups (*P* > 0.05). Subgroup analysis concerning ovarian stimulation protocol indicated that in the subgroup of gonadotrophin-releasing hormone (GnRH) antagonist protocol, the clinical pregnancy rate of the non-vitamin D deficiency group was significantly higher than that of the other two groups (*P* < 0.05).

**Conclusion:**

Serum vitamin D level was not associated with embryo quality and pregnancy outcomes for patients with NOR. Further studies with greater sample sizes and a longer follow-up period are needed to elucidate the relationships between vitamin D levels and IVF outcomes.

## 1 Introduction

Vitamin D, a vital lipid-soluble steroid hormone, is primarily synthesized through the exposure of the skin to ultraviolet B radiation and dietary intake. It plays a crucial role in various biological processes, including cell differentiation, maturation, and the innate immune system, which are mediated by the vitamin D receptor (VDR) (1). Over the years, a wealth of clinical evidence has shown that vitamin D deficiency is prevalent in different countries and regions around the world and has been highly suspected to be related to some chronic diseases or acute conditions (2). Consequently, Physicians have increasingly emphasized the importance of vitamin D testing, particularly among children and pregnant women, in order to address its impact on human health. The serum concentration of 25-hydroxyvitamin D (25OH-D), commonly considered to be the best indicator of vitamin D status in the human body, has been applied to guide the establishment of appropriate vitamin D dietary and therapeutic requirements for patients with vitamin D deficiency.

Contemporary society and environmental changes have contributed to a rising incidence of infertility, prompting the widespread utilization of assisted reproductive technology (ART) on a global scale (3, 4). Despite significant advancements in ovarian stimulation protocols and embryo laboratory techniques over the last decade, the progress in improving pregnancy outcomes following embryo transfer (ET) has encountered a plateau. To address this challenge, researchers are striving to explore novel therapeutic approaches and investigate the impact of specific nutrient deficiencies, such as vitamin D, in optimizing ART outcomes.

A number of experimental studies have demonstrated the presence of vitamin D receptors (VDR) in various reproductive organs, including ovarian granulosa cells and endometrium, suggesting a close association between vitamin D and female reproductive potential (5). However, no consensus on the relationship between vitamin D and ART outcomes has been reached based on published research findings. Many studies have reported that the possibility of achieving clinical pregnancy after ET was higher in patients with adequate vitamin D and vitamin D supplementation may improve ART outcomes and diminished ovarian reserve (DOR), endometrial function, and even ameliorate insulin resistance in patients with polycystic ovary syndrome (PCOS) (6–10), whereas some studies indicated that the level of serum vitamin D has no effect on the chances of conception in both ovulation induction and ET cycles (11–13). It should be noted that the above studies primarily focused on patients with PCOS or DOR and ignored the concerns of patients with normal ovarian reserve (NOR) who generally have a favorable prognosis. Given the conflicting evidence described above and the scant attention given to patients with NOR, the aim of the present study was to evaluate whether serum vitamin D affects the clinical outcomes of ART in patients with NOR.

## 2 Materials and methods

### 2.1 Study population and design

This study was approved by the institutional review board of the Affiliated Taizhou People’s Hospital to Nanjing Medical University (Approval Number: KY 2023-076-01). This retrospective cohort study was conducted at the Department of reproductive medicine of the hospital from September 2021 to March 2023. Follow-up of clinical outcomes from the initial ET was completed in June 2023. A total of 264 patients who completed their first IVF/ICSI cycle and underwent the fresh ET cycle 3-6 days after oocyte retrieval or the first frozen-thawed ET (FET) cycle within 3 months after applying the freeze-all protocol were included in this study. The following exclusion criteria were applied (1): patients who had not undergone 25OH-D measurements at the beginning of ovulation induction therapy (2); patients aged 40 years or older; (3) patients who chose to delay their first ET to 3 months after oocyte retrieval (these patients may have obvious fluctuations in 25OH-D levels); (4) patients taking vitamin D supplements; (5) patients suffering from DOR (antral follicle count [AFC] < 5, anti-mullerian hormone [AMH] < 1.2 ng/mL) (14), PCOS based on Rotterdam criteria (which require the presence of at least two of the following: i. oligo- or anovulation; ii. clinical and/or biochemical hyperandrogenism after excluding other potential causes; iii. polycystic ovaries detected via ultrasound) (15), chromosomal abnormalities, hydrosalpinx, uterine malformation, precancerous lesions and malignant neoplasm; (6) preimplantation genetic testing (PGT) cycles; (7) egg donation cycles.

Serum 25OH-D was measured with the 25-OH Vitamin D Reagent Kit on the Abbott Architect i2000SR Immunoassay Analyzer (Abbott Diagnostics). The detection range of the kit is 3.4 - 155.9 ng/ml. According to the Institute of Medicine (IOM) and the Endocrine Society clinical practice guidelines (16, 17), 25OH-D values were stratified into three groups: severe vitamin D deficiency group (25OH-D < 10 ng/ml), vitamin D deficiency group (10 ng/ml ≤ 25OH-D < 20 ng/ml), and non-vitamin D deficiency group (25OH-D ≥ 20 ng/ml).

### 2.2 Procedures

Patients were treated with the conventional ovarian stimulation protocols, including long gonadotrophin-releasing hormone (GnRH) agonist protocol, GnRH antagonist protocol, and progestin-primed ovarian stimulation (PPOS) protocol. The specific protocol applied and the initial dose of gonadotrophin (Gn) (recombinant follicle stimulating hormone [rFSH] alone or in combination with urinary human menopausal gonadotropin [hMG]) was decided by the patient’s characteristics and physicians’ preferences. The majority of patients undergoing their initial ovulation induction therapy adopted the most commonly employed GnRH agonist protocol or GnRH antagonist protocol, and a minority of patients, due to economic constraints, pursued the PPOS protocol after thorough consultation with their physicians. For the long GnRH agonist protocol and GnRH antagonist protocol, pituitary suppression was achieved by either injection of GnRH agonist (Leuprorelin Acetate Microspheres Sustained Release For Injection; Beijing Biote Pharmaceutical Co., Ltd.) starting in the early follicular phase or the injection of GnRH antagonist (Ganirelix Acetate Injection; Nanjing Zhengda Tianqing Pharmaceutical Co., Ltd.) when at least one follicle over 12mm, while the adjustment of follicle growth rate was realized by increasing or decreasing the dose of rFSH (Gonal-F; Merck Serono S.p.A.) and hMG (Menotrophins for Injection; Livzon (GROUP) Pharmaceutical Co., Ltd). For the PPOS protocol, medroxyprogesterone acetate (Medroxyprogesterone Acetate Tablets; Xianju Pharmaceutical Co., Ltd.) combined with Gn was administered on day 3 of the menstrual cycle until trigger day. When 2 or more follicles reached 18 mm in diameter, human chorionic gonadotropin (hCG) (Chorionic Gonadotropin For Injection; Livzon (GROUP) Pharmaceutical Co., Ltd.) or GnRH agonist (Decapeptyl; Ferring AG) were administered individually or in combination to induce eventual oocyte maturation according to physician preference and the presence of ovarian hyperstimulation syndrome (OHSS) in patients. Oocytes retrieval was conducted 36 h later. Oocyte fertilization was carried out by conventional insemination or intracytoplasmic sperm injection (ICSI) depending on semen parameters. Two experienced embryologists identified normal fertilization if two distinct pronuclei (2PN) were displayed in the embryo. The available cleavage embryos were defined as grade III and above according to the Istanbul consensus (18), and available blastocysts had a score of 3BC or above according to the Gardner morphological criteria (19).

Fresh ET was performed 3 or 5 days after oocyte retrieval if patients who were willing to undergo it had qualified endometrium (> 7 mm), a low risk of OHSS, and unelevated progesterone (P) on trigger day. Other patients underwent the first frozen-thawed ET (FET) cycle within the next three months, using a natural or artificial protocol for endometrium preparation. Luteal phase support was achieved by 40mg of intramuscular progesterone (Progesterone Injection; Tianjin Kingyork Pharmaccuticals Co.) and 40 mg of oral dydrogesterone (Duphaston; Abbott Biologicals B.V.) in both fresh and frozen ET cycles. A maximum of 2 embryos transferred per cycle was permitted.

### 2.3 Outcome measurements

The primary outcome of this study was clinical pregnancy defined as the presence of an intrauterine gestational sac with fetal heartbeat detected 28–35 days after ET. The secondary outcomes were mature oocytes (MII), normal fertilization, available cleavage embryo, available blastocyst, biochemical pregnancy defined as a positive serum β-HCG result 14 days after ET, early abortion defined as the loss of recognized clinical pregnancy before 12 weeks of gestation, and embryo implantation rate defined as the total number of gestational sacs divided by the number of embryos transferred.

### 2.4 Statistical analysis

All statistical analyses were performed using the Statistical Package for Social Sciences (SPSS) 27.0 (IBM Corp., USA) and figures were drawn by Graph Pad Prism 9.5.1 (GraphPad Software, LLC., USA). Continuous data were represented as medians and inter-quartile range (IQR, 25th–75th percentile) and analyzed by Kruskal–Wallis test since all data showed abnormal distribution. Categorical data were represented as frequency and percentage and analyzed by Pearson’s chi-square test or Fisher’s exact test, as appropriate. In case of significant differences among the three groups, Bonferroni tests were conducted as *post-hoc* tests to identify which two groups of data present statistical differences.

For the binary variable outcomes, we used a modified Poisson regression model to calculate relative risk (RR) which can show a more accurate relationship between exposure (serum 25OH-D level) and outcome (pregnancy outcomes) compared with the odds ratio (OR) obtained from logistic regression analysis, since the incidence of pregnancy outcomes is greater than 10% (20). For the continuous variable outcomes, we performed multiple linear regression analyses to assess the correlation between 25OH-D and outcomes. Potential confounders which were significantly less than 0.10 analyzed by univariable analyses were selected into the regression models. Also, age, the number of embryos transferred, and the season of blood sampling or ET were included in the models based on clinical or theoretical experience. Before constructing the regression model, the Pearson correlation matrix was used to evaluate the collinearity of variables. If the correlation of any variable pair is greater than 0.70, there is collinearity between the two variables and only one of them was included in the regression model. Crude and adjusted RRs or β with 95% confidence intervals (CIs) for outcomes were recorded. To evaluate the robustness of the result of the primary outcome, subgroup analyses according to the season of ET, phase of embryo transferred, endometrial thickness, and ovarian stimulation protocol and sensitivity analysis using a conventional logistic regression model with the same covariables used in the modified Poisson model were performed. The cut-off value of endometrial thickness for subgroup analysis was determined by the receiver operating characteristic (ROC) curve. The PPOS protocol within the ovarian stimulation protocol, with only thirteen cycles, was deemed inadequate for conducting multivariate analysis and, as a result, subgroup analysis was not performed.

Before processing data, seasons were defined according to the calendar definition for China: Spring: March 1 to May 31; Summer: June 1 to August 31; Autumn: September 1 to November 30; Winter: December 1 to February 28. *P*-values less than 0.05 were considered statistically significant.

## 3 Results

### 3.1 Baseline characteristics

**Figure 1:**
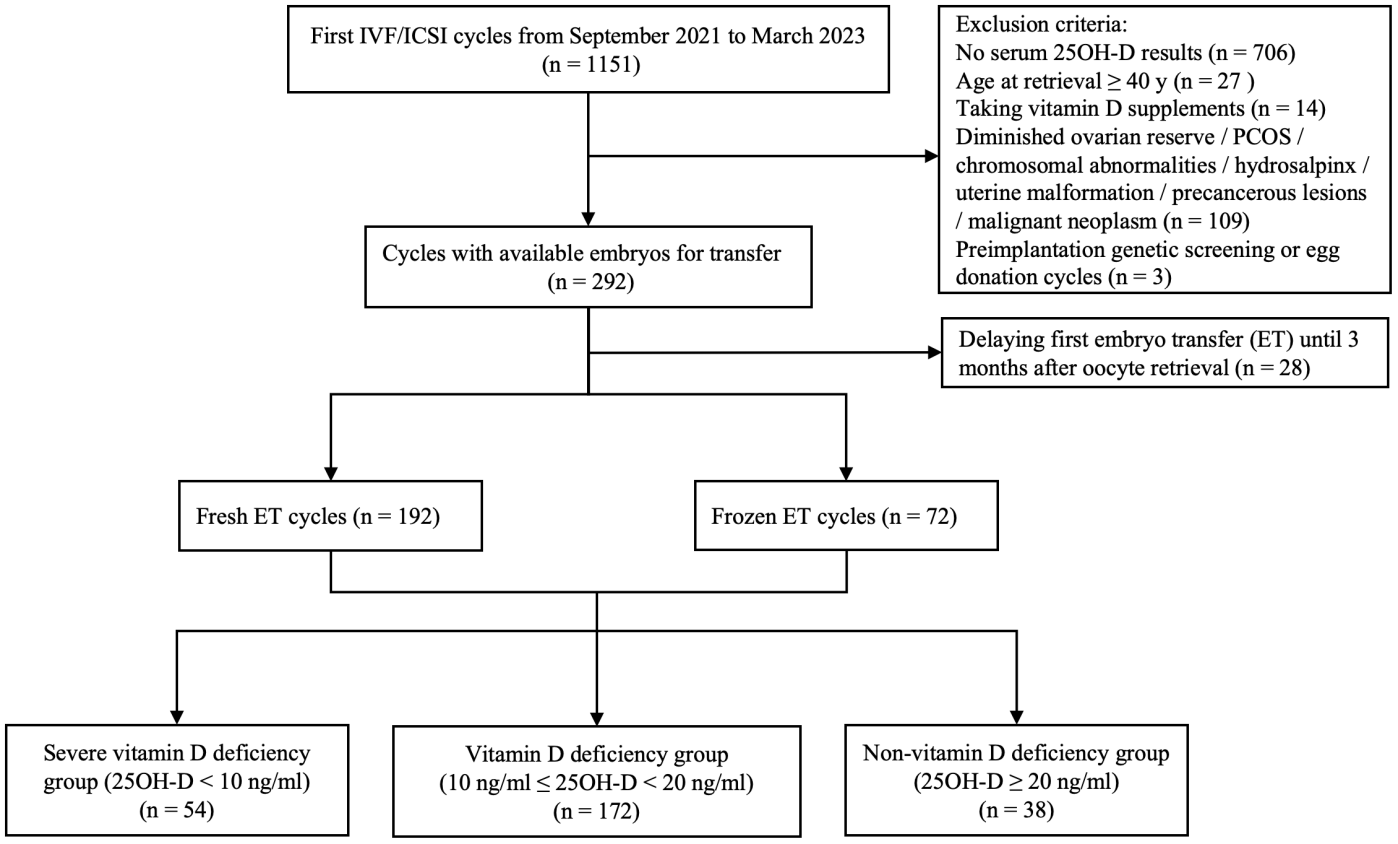
Patient selection process.

A total of 264 patients who were satisfied with the selection criteria were included in the final analysis (**Figure 1**). Among all patients, the serum 25OH-D level was 13.1 (10.5, 17.9) ng/ml. 20.5% (n = 54) were severely vitamin D deficient, 65.2% (n = 172) were vitamin D deficient, while the remaining patients (n = 38) had no vitamin D deficiency. As expected, there were significantly more patients without vitamin D deficiency than those with severe vitamin D deficiency in summer and autumn (*P* = 0.001 and 0.012, respectively). In spring, severe vitamin D deficiency was more prevalent than non-vitamin D deficiency (*P* = 0.007). In winter, patients with severe vitamin D deficiency and vitamin D deficiency were more than those without vitamin D deficiency (*P* = 0.001 and 0.007, respectively) (**Table 1**).

**Table 1:** Comparison of baseline characteristics among three groups with different vitamin D levels.

Characteristic	25OH-D < 10 ng/ml	10 ng/ml ≤ 25OH-D < 20 ng/ml	25OH-D ≥ 20 ng/ml	*P* Value
Number	54	172	38	
Age (years)	28 (26, 32)	30 (27, 33)	30 (28, 33)	0.082 ^a^
Infertility duration (years)	2 (1, 3)	2 (1, 4)	3 (2, 4)	0.142 ^a^
Season of blood drawing (%)				0.000 ^b^
Spring	21 (38.9)	46 (26.7)	5 (13.2) ^c^	
Summer	6 (11.1)	45 (26.2)	15 (39.5) ^c^	
Autumn	11 (20.4)	45 (26.2)	17 (44.7) ^c^	
Winter	16 (29.6)	36 (20.9)	1 (2.6) ^d^	
BMI (kg/m2)	21.50 (19.95, 25.15)	22.95 (20.83, 27.25)	22.55 (20.80, 25.55)	0.109 ^a^
Basal FSH (IU/L)	6.42 (5.43, 8.11)	6.21 (5.19, 7.62)	6.43 (5.27, 7.78)	0.538 ^a^
Basal LH (IU/L)	4.20 (3.25, 7.03)	4.61 (2.94, 6.32)	4.09 (2.54, 6.17)	0.664 ^a^
Basal E_2_ (pg/ml)	30.90 (24.79, 41.86)	31.59 (24.33, 42.69)	27.98 (22.74, 43.79)	0.833 ^a^
AMH (ng/ml)	3.49 (2.61, 6.20)	3.89 (2.41, 5.73)	3.85 (1.93, 6.23)	0.929 ^a^
AFC	18 (14, 21)	16 (13, 22)	18 (12, 22)	0.784 ^a^
Primary etiology				0.828 ^b^
Tubal factor	19 (35.2)	72 (41.9)	16 (42.1)	
Ovulatory dysfunction	9 (16.7)	30 (17.4)	4 (10.5)	
Endometriosis	3 (5.6)	9 (5.2)	3 (7.9)	
Uterine factor	2 (3.7)	2 (1.2)	2 (5.3)	
Sperm factor	8 (14.8)	23 (14.0)	5 (13.2)	
Unexplained fertility	13 (24.1)	35 (20.3)	8 (21.1)	
Gravidity				0.580 ^b^
0	37 (68.5)	100 (58.1)	22 (57.9)	
1	10 (18.5)	40 (23.3)	7 (18.4)	
≥ 2	7 (13.0)	32 (18.6)	9 (23.7)	
Parity				0.145 ^b^
0	49 (90.7)	140 (81.4)	27 (71.1)	
1	5 (9.3)	29 (16.9)	10 (26.3)	
≥ 2	0 (0.0)	3 (1.7)	1 (2.6)	
Ovarian stimulation protocol				0.136 ^b^
GnRH agonist	32 (59.3)	82 (47.7)	22 (57.9)	
GnRH antagonist	22 (40.7)	80 (46.5)	13 (34.2)	
PPOS	0 (0.0)	10 (5.8)	3 (7.9)	
Duration of stimulation (days)	11 (9, 11)	10 (9, 12)	11 (9, 12)	0.441 ^a^
Total dose of Gn (IU)	2213 (1800, 2700)	2194 (1763, 2859)	2063 (1650, 2719)	0.755 ^a^
LH on HCG day (IU/L)	0.91 (0.57, 1.48)	1.03 (0.60, 1.95)	1.26 (0.63, 2.32)	0.104 ^a^
E_2_ on HCG day (pg/mL)	2158.67 (1122.24, 4177.34)	1997.56 (1333.32, 3190.03)	1883.57 (1389.50, 3581.07)	0.784 ^a^
P on HCG day (ng/mL)	0.80 (0.52, 1.04)	0.73 (0.53, 1.10)	0.92 (0.50, 1.14)	0.485 ^a^
Fertilization method				0.826 ^b^
IVF	44 (81.5)	135 (78.5)	29 (76.3)	
ICSI	10 (18.5)	37 (21.5)	9 (23.7)	

a Kruskal-Wallis test followed by a post-hoc pairwise comparison.

b Chi-square test followed by Bonferroni post-hoc test.

c statistically significant differences from the severe vitamin D deficiency group.

d statistically significant differences from the severe vitamin D deficiency group and the vitamin D deficiency group.

25OH-D, 25-hydroxyvitamin D; BMI, body mass index; FSH, follicle stimulating hormone; LH, luteinizing hormone; E_2_, estradiol; AFC, antral follicle count; AMH, anti-mullerian hormone; GnRH, gonadotrophin-releasing hormone; PPOS, progestin-primed ovarian stimulation; Gn, gonadotrophin; HCG, human chorionic gonadotropin; P, progesterone; IVF, in vitro fertilization; ICSI, intracytoplasmic sperm injection

There were no significant differences in age, body mass index (BMI), basal FSH, basal luteinizing hormone (LH), basal E_2_, AMH, AFC, etiology, gravidity, and parity among the three groups (*P* > 0.05). Also, the ovarian stimulation protocol, duration of stimulation, the total dose of Gn, LH on HCG day, E_2_ on HCG day, P on HCG day, and fertilization method were similar among the three groups (*P* > 0.05) (**Table 1**).

### 3.2 Primary outcome

**Figure 2:**
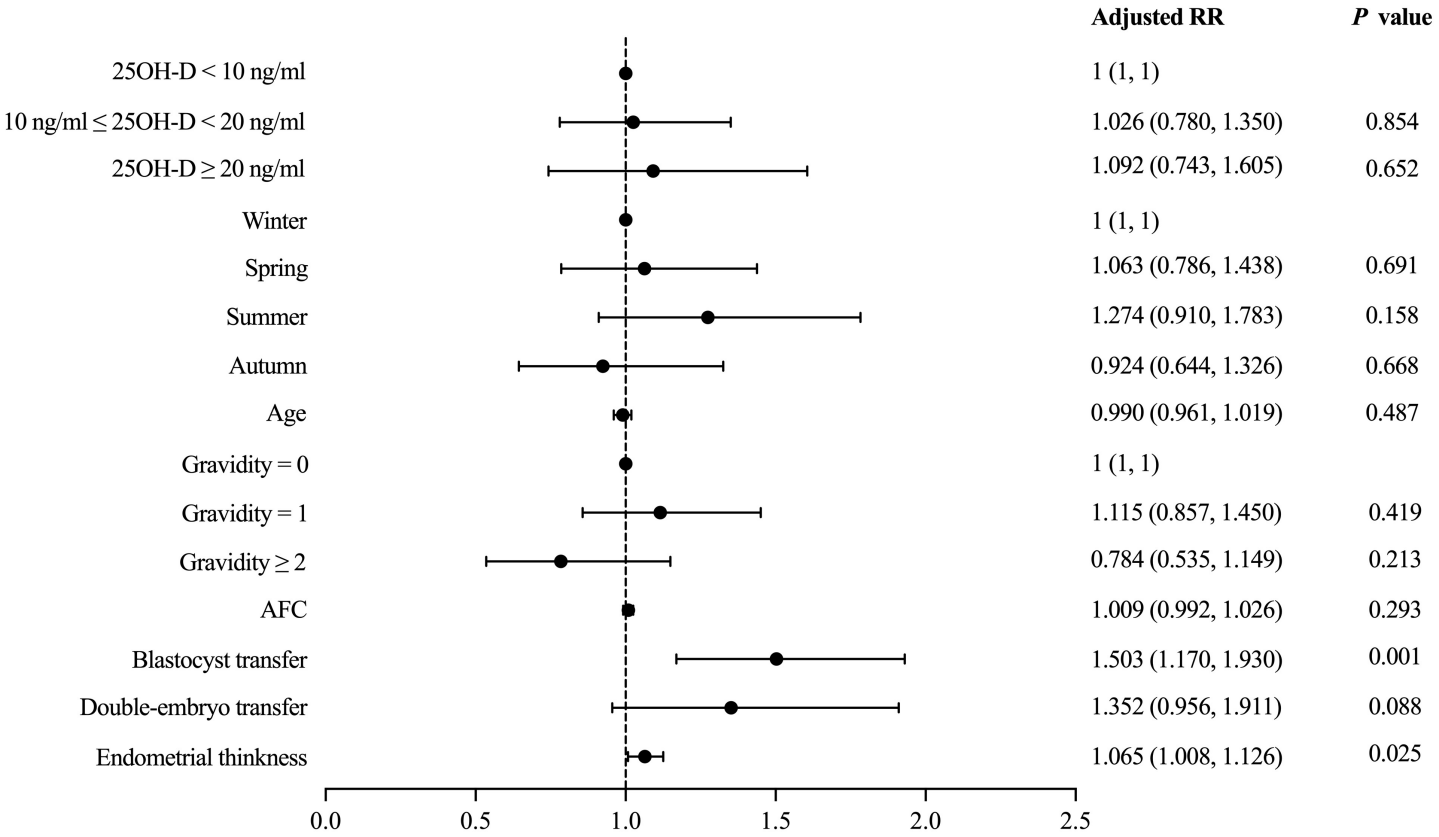
Modified Poisson regression model evaluating the effect of covariates on the primary outcome.

Vitamin D grouping was not associated with clinical pregnancy (51.9% vs. 54.1% vs. 52.6%, *P* = 0.955) (**Table 2**). The results of the univariate analysis for clinical pregnancy are summarized in **Supplementary Table 1**. Overall, 8 independent variables (25OH-D grouping, season of ET, age, AFC, gravidity, phase of embryo transferred, number of embryos transferred, endometrial thickness on [theoretical] ovulation triggering day) that may be associated with clinical pregnancy were included in the modified Poisson regression model. The final regression model shows that the main factors associated with clinical pregnancy were the phase of embryo transferred (adjusted RR = 1.503; 1.170 - 1.930; *P* = 0.001) and endometrial thickness (adjusted RR = 1.065; 1.008 – 1.126; *P* = 0.025) (**Figure 2**). Vitamin D grouping (vitamin D deficiency group: adjusted RR = 1.026; 0.780 - 1.350; *P* = 0.854; non-vitamin D deficiency group: adjusted RR = 1.092; 0.743 - 1.605; *P* = 0.652) was not individualized as an independent covariate for the prediction of clinical pregnancy (**Table 3**, **Figure 2**).

**Table 2:** Comparison of primary and secondary outcomes among three groups with different vitamin D levels.

Outcomes	25OH-D < 10 ng/ml (n = 54)	10 ng/ml ≤ 25OH-D < 20 ng/ml (n = 172)	25OH-D ≥ 20 ng/ml (n = 38)	*P* Value
Primary outcome				
Clinical pregnancy rate (%)	28 (51.9)	93 (54.1)	20 (52.6)	0.955 ^b^
Secondary outcomes				
No. of MII oocyte	9 (6, 16)	10 (7, 14)	8 (6, 13)	0.318 ^a^
No. of 2PN	6 (3, 9)	6 (4, 10)	6 (4, 9)	0.838 ^a^
No. of available cleavage embryo	4 (2, 8)	4 (2, 7)	4 (2, 7)	0.823 ^a^
No. of available blastocyst	1 (0, 3)	1 (0, 3)	0 (0, 2)	0.085 ^a^
Biochemical pregnancy rate (%)	30 (55.6)	97 (56.4)	23 (60.5)	0.878 ^b^
Early abortion rate (%)	3 (10.7)	10 (10.8)	6 (28.6)	0.097 ^b^
Embryo implantation rate (%)	42 (42.9)	118 (39.0)	22 (30.6)	0.261 ^b^

a Kruskal-Wallis test followed by a post-hoc pairwise comparison.

b Chi-square test followed by Bonferroni post-hoc test.

25OH-D, 25-hydroxyvitamin D; MII, mature oocytes; 2PN, 2 pronuclear.

**Table 3:** Multivariable analyses evaluating the relationship between different vitamin D groups and multiple outcomes.

Outcomes	25OH-D < 10 ng/ml (n = 54)	10 ng/ml ≤ 25OH-D < 20 ng/ml (n = 172)	*P* value	25OH-D ≥ 20 ng/ml (n = 38)	*P* value
Primary outcome					
Clinical pregnancy	28 (51.9)	93 (54.1)		20 (52.6)	
Crude RR (95%*CI*)	1	1.043 (0.779, 1.396)	0.778	1.015 (0.683, 1.509)	0.941
Adjusted RR (95%*CI*)	1	1.026 (0.780, 1.350)	0.854	1.092 (0.743, 1.605)	0.652
Secondary outcome					
MII oocyte	9 (6, 16)	10 (7, 14)		8 (6, 13)	
Crude β (95%*CI*)	1	-1.071 (-3.081, 0.938)	0.295	-1.292 (-2.865, 0.281)	0.106
Adjusted β (95%*CI*)	1	-0.443 (-2.337, 1.451)	0.646	-0.735 (-2.441, 0.972)	0.394
2PN	6 (3, 9)	6 (4, 10)		6 (4, 9)	
Crude β (95%*CI*)	1	-0.827 (-2.383, 0.730)	0.296	-0.536 (-1.804, 0.733)	0.404
Adjusted β (95%*CI*)	1	-0.329 (-1.809, 1.151)	0.662	0.022 (-1.408, 1.452)	0.976
Available cleavage embryo	4 (2, 8)	4 (2, 7)		4 (2, 7)	
Crude β (95%*CI*)	1	-0.547 (-1.778, 0.683)	0.382	-0.411 (-1.360, 0.538)	0.392
Adjusted β (95%*CI*)	1	-0.155 (-1.361, 1.051)	0.800	-0.001 (-1.0865, 1.083)	0.998
Available blastocyst	1 (0, 3)	1 (0, 3)		0 (0, 2)	
Crude β (95%*CI*)	1	-0.542 (-1.392, 0.307)	0.210	-0.453 (-1.193, 0.287)	0.227
Adjusted β (95%*CI*)	1	-0.412 (-1.243, 0.419)	0.329	-0.424 (-1.172, 0.323)	0.262
Biochemical pregnancy	30 (55.6)	97 (56.4)		23 (60.5)	
Crude RR (95%*CI*)	1	1.015 (0.773, 1.333)	0.914	1.089 (0.767, 1.547)	0.632
Adjusted RR (95%*CI*)	1	1.000 (0.773, 1.294)	0.998	1.176 (0.839, 1.647)	0.347
Early abortion	3 (10.7)	10 (10.8)		6 (28.6)	
Crude RR (95%*CI*)	1	1.004 (0.297, 3.396)	0.995	2.667 (0.753, 9.450)	0.129
Adjusted RR (95%*CI*)	1	0.996 (0.310, 3.202)	0.995	2.462 (0.782, 7.749)	0.124
Embryo implantation	42 (42.9)	118 (38.3)		22 (30.6)	
Crude RR (95%*CI*)	1	0.894 (0.683, 1.170)	0.414	0.713 (0.470, 1.081)	0.111
Adjusted RR (95%*CI*)	1	0.870 (0.668, 1.133)	0.301	0.777 (0.513, 1.176)	0.232

25OH-D, 25-hydroxyvitamin D; RR, risk ratio; CI, confidence interval; MII, mature oocytes; 2PN, 2 pronuclear.

### 3.3 Secondary outcomes

No significant differences were observed among three vitamin D groups regarding the number of MII oocytes (9 [6, 16] vs. 10 [7, 14] vs. 8 [6, 13], *P* = 0.318), number of 2PN (6 [3, 9] vs. 6 [4, 10] vs. 6 [4, 9], *P* = 0.838), number of available cleavages embryo (4 [2, 8] vs. 4 [2, 7] vs. 4 [2, 7], *P* = 0.823), number of available blastocysts (1 [0, 3] vs. 1 [0, 3] vs. 0 [0, 2], *P* = 0.085), biochemical pregnancy rate (55.6% vs. 56.4% vs. 60.5%, *P* = 0.878), early abortion rate (10.7% vs. 10.8% vs. 28.6%, *P* = 0.097), and implantation rate (42.9% vs. 39.0 vs. 30.6%, *P* = 0.261) (**Table 2**). For the continuous variable outcomes, adjustment was performed according to the following cofounders: 25OH-D grouping, the season of blood sampling, age, AFC, gravidity, and parity (**Supplementary Table 2**). AMH was not included in the linear regression model because of the collinearity between AMH and AFC. The results of multiple linear regression showed that vitamin D grouping had no significant correlation with the number of MII oocytes, number of 2PN, number of available cleavage embryos, and number of available blastocysts (*P* > 0.05) (**Table 3**). For the binary variable outcomes, adjustment was conducted according to the potential confounders of clinical pregnancy. The results of the modified Poisson regression model revealed that there were no significant associations in biochemical pregnancy rate, early abortion rate, and implantation rate in when severe vitamin D deficiency group compared with the vitamin D deficiency group and non-vitamin D deficiency group, respectively (*P* > 0.05) (**Table 3**).

### 3.4 Exploratory subgroup analyses

**Figure 3:**
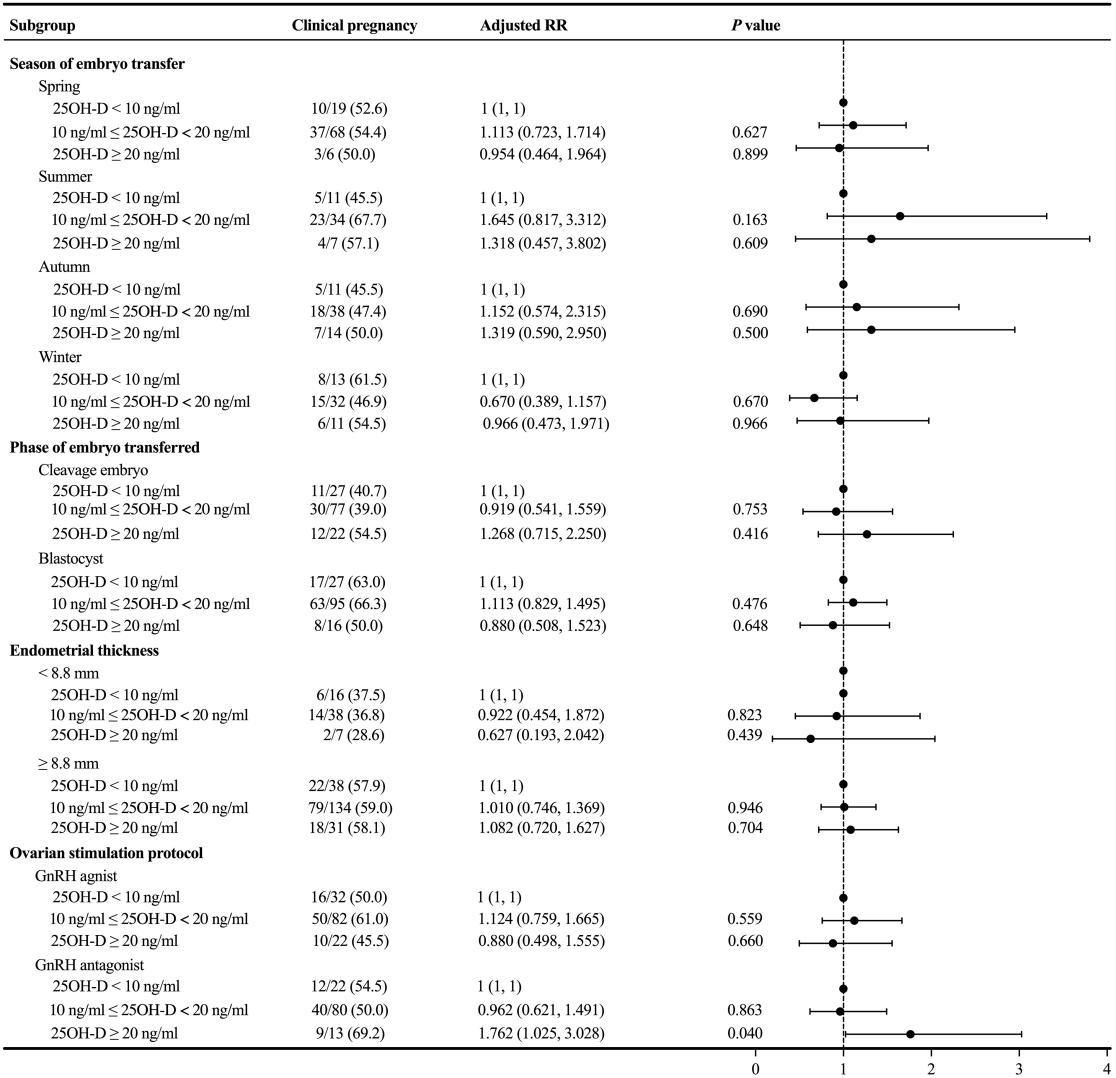
Subgroup analyses evaluating the relationship between different vitamin D groups and the primary outcome.

Because there was no significant association between the degree of vitamin D deficiency and the primary outcome, we performed subgroup analyses to further explore whether there was a difference in clinical pregnancy rate in a particular subgroup. In the subgroup analyses concerning the season of ET, phase of embryo transferred, and endometrial thickness, the probability of clinical pregnancy was similar among the three groups with different levels of serum 25OH-D (*P* > 0.05) (**Figure 3**). In the subgroup analysis concerning the ovarian stimulation protocols, following adjustment for relevant confounding factors, the clinical pregnancy rate among patients in the GnRH antagonist subgroup without vitamin D deficiency was significantly higher than in both the severe vitamin D deficiency group (adjusted RR = 1.762; 1.025 – 3.028; *P* = 0.040) and the vitamin D deficiency group (Ref: vitamin D deficiency group; adjusted RR = 1.735; 1.059 – 2.843; *P* = 0.029).

### 3.5 Sensitivity analysis

The result of the logistic regression model still indicated that thicker endometrium (adjusted OR = 1.157; 1.006 - 1.330, *P* = 0.041) and transferring blastocyst (adjusted OR = 2.486; 1.446 – 4.274, *P* = 0.001) were associated with a higher likelihood of clinical pregnancy for patients with normal ovarian reserve, and no significant correlation was shown between different vitamin D groups and clinical pregnancy (*P* > 0.05) (**Table 4**).

**Table 4:** Multivariable logistic regression evaluating the effect of covariates on primary outcome.

Parameter	β	SE	Wald χ2	Adjusted OR (95%CI)	*P* value
25OH-D group					
25OH-D < 10 ng/ml	Reference				
10 ng/ml ≤ 25OH-D < 20 ng/ml	0.080	0.334	0.057	1.083 (0.563, 2.083)	0.811
25OH-D ≥ 20 ng/ml	0.209	0.458	0.209	1.233 (0.503, 3.023)	0.648
Season of embryo transfer					
Winter	Reference				
Spring	0.171	0.368	0.217	1.187 (0.577, 2.442)	0.641
Summer	0.609	0.423	2.073	1.838 (0.803, 4.212)	0.150
Autumn	-0.169	0.394	0.183	0.845 (0.390, 1.830)	0.669
Age	-0.024	0.036	0.448	0.976 (0.910, 1.047)	0.503
Gravidity					
0	Reference				
1	0.259	0.342	0.573	1.296 (0.663, 2.533)	0.449
2	-0.434	0.402	1.169	0.648 (0.295, 1.424)	0.280
AFC	0.022	0.022	0.965	1.022 (0.978, 1.068)	0.326
Blastocysts transfer cycle	0.911	0.276	10.855	2.486 (1.446, 4.274)	0.001
Double-embryo transfer cycle	0.632	0.349	3.272	1.882 (0.949, 3.732)	0.070
Endometrial thickness	0.146	0.071	4.193	1.157 (1.006, 1.330)	0.041

25OH-D, 25-hydroxyvitamin D; AFC, antral follicle count.

## 4 Discussion

With vitamin D testing increasing globally, particularly in cases of infertility, it is critical to consider if we are prescribing unnecessary medical examination and excessive medical treatment leading to the waste of health-care resources and unnecessary concern for patients. In this retrospective cohort study, our results indicate that there were no significant associations between vitamin D levels and multiple ART outcomes in patients with normal ovarian reserve undergoing the first IVF/ICSI cycles. 25OH-D levels showed expected seasonal variation, with fewer individuals displaying vitamin D deficiency in summer and autumn than in spring and winter. The findings may provide preliminary evidence that there is no need to be overly concerned about serum vitamin D levels in NOR patients with good prognoses.

One important speculation about the important role of vitamin D in assisted reproduction is that it may fundamentally improve ovarian reserve, promote ovulation, and influence embryo development. PCOS stands as the foremost cause of anovulatory infertility, resulting in reduced fertility potential, alongside an elevated risk of perinatal complications (21, 22). Previous studies investigating the association between serum vitamin D levels and ovulation rate have reported positive findings in patients with PCOS. Several studies showed that patients with both vitamin D deficiency and PCOS were less likely to ovulate in ovarian stimulation cycles (23, 24), and taking vitamin D supplementation for patients with PCOS undergoing ovulation induction could improve ovulation rate (8, 12). This improvement may be attributed to sufficient vitamin D can increase the soluble receptor for advanced glycation end-products (AGEs) which can bind circulating AGEs and have an anti-inflammatory effect in PCOS patients (25, 26). Additionally, folliculogenesis in PCOS may be enhanced by vitamin D since the abnormally increased AMH levels can be decreased by vitamin D supplementation (27–29). The benefits of adequate vitamin D can also be seen in enhanced fertilization rates during *in vitro* fertilization (IVF) for PCOS patients (30). However, differences in clinical pregnancy rates and live birth rates after ovulation induction or embryo transfer were inconsistent among groups with different vitamin D levels (8, 12, 23). There are also some investigations focusing on the role of vitamin D in patients with DOR with disparate findings. While one single-arm uncontrolled study supported a possible favorable effect of vitamin D on increased AMH expression, a retrospective cohort study showed that AMH and FSH levels did not differ between DOR patients with low and normal vitamin D levels (9, 31). In the present study, the results showed that vitamin D levels did not affect the development of oocytes and embryos in NOR patients since the three groups with different levels of vitamin D shared a similar number of mature oocytes, 2PN embryos, available cleavage embryos, and blastocysts. Given the uncertain findings, it would be illogical to recommend vitamin D supplementation in patients having preserved ovarian reserve.

Another significant hypothesis around the crucial role of vitamin D in ET is that it can affect endometrial receptivity. The expression of VDR in the endometrium or uterus and the differences in VDR expression between the proliferative and secretory phases suggest that vitamin D may modify endometrial receptivity (32). Also, patients with sufficient vitamin D expressed more HOXA10 mRNA in the endometrium which plays a crucial part in making the endometrium more receptive for embryo implantation through promoting the decidualization of endometrial epithelial cells and improving local immunomodulation (33, 34). However, clinical data showed insignificant differences in pregnancy rates that did not meet experimental expectations among groups with different vitamin D levels. To our knowledge, four meta-analyses have examined whether vitamin D level was related to the clinical outcomes of IVF with varying conclusions (6, 13, 35, 36), whereas two meta-analyses assessing the relationship between vitamin D supplementation and ART outcomes suggested that using vitamin D may be effective in improving chemical pregnancy rate or clinical pregnancy rate (7, 37). The findings of a two-center randomized superiority double-blind placebo-controlled trial conducted in Italy showed that a single dose of 600,000 IU of vitamin D3 did not improve the clinical pregnancy rate and concluded that routine vitamin D supplementation in patients with a preserved ovarian reserve and vitamin D deficiency was not supported (38). The findings of our study align with the results of the latest meta-analysis of 14 studies showing no significant correlation between different vitamin D groups and pregnancy outcomes after ET (13). Even most subgroup analyses concerning the clinical pregnancy in this study were unable to identify any condition for which vitamin D supplementation could be beneficial. Although one subgroup analysis pertaining to the GnRH antagonist protocol suggested a favorable effect of normal 25OH-D levels, the clinical pregnancy rate in the vitamin D deficiency group was lower than that in the severe vitamin D deficiency group. The elevation of serum vitamin D levels did not exhibit a consistent upward trend in clinical pregnancy rates, thus rendering the results inconclusive for guiding clinical practice. This observation could potentially be attributed to the limitations imposed by the constrained sample size. As expected, blastocyst transferring and a thicker endometrial improve the CPR after ET, as the culture process of blastocyst provides more morphogenetic information to identify and discard embryos with lower implantation potential, while blastocyst and endometrial of moderate thickness also promote better embryo-endometrial synchrony (39, 40).

The season is an important time division that guides people’s life artificially formulated according to the variations of climate, temperature, and the number of daylight hours. Since the production of serum vitamin D mainly depends on the synthesis of cholecalciferol in the skin through sun exposure, the season is an important factor affecting serum vitamin D. It is known that season may have an impact on the natural conception and live birth, one of the main reasons for this is because vitamin D levels increase during summer and autumn (41), but results of the correlation between seasons and ART outcomes are inconsistent. A recently published study even shows that live births are significantly lower in summer compared to spring and autumn, and similar clinical pregnancy probabilities were seen between summer and winter (42). In this study, to avoid seasons from interfering with the multiple outcome analysis, we included the season of blood sampling and the season of ET in the multivariate analysis of embryo development and ET outcomes, respectively. Remarkably, our findings revealed that neither vitamin D levels nor season had a significant effect on these outcomes. Meantime, despite the widespread prevalence of vitamin D deficiency around the world, the fact that only a minority population is diagnosed with infertility indicates that vitamin D may not play a substantial role in infertility. Our results indirectly reflect the physiological changes associated with seasonal variations have minimal impact on assisted reproduction, allowing patients to choose their preferred admission time.

In this study, despite observing slightly inferior outcomes in patients with non-deficient vitamin D levels compared to those with sufficient vitamin D levels, it remains inconclusive to establish a direct association between decreased vitamin D levels and improved reproductive outcomes. This is because the differences among the three groups were not statistically significant, and it is plausible that the limited sample size may have contributed to unstable results and reduced statistical power.

The main strength of our study is the consideration of NOR patients with good prognoses, thereby generating multiple outcomes compensating for the lack of data in this area in existing studies. Besides, both embryo quality and clinical outcomes among various vitamin D groups were analyzed, allowing for a more comprehensive evaluation of the role of vitamin D in ART. Furthermore, we innovatively employed modified Poisson regression analysis adjusting for potential confounders to obtain RR values during multivariate analysis which can more accurately reflect the relationship between vitamin D levels and binary outcomes. However, there are several limitations to our study. The main limitation is its retrospective design, which inherently lacks a prospective nature. In addition, the credibility of the results was restricted by the small number of patients in three groups (only 264 patients satisfied the selection criteria). Moreover, no live birth rates were reported in our study due to the limited follow-up time, and no further research on vitamin D supplementation was conducted.

## 5 Conclusions

In summary, our results indicate that there were no significant differences observed in the number of mature oocytes, 2PN embryos, available cleavage embryos, available blastocysts, biochemical pregnancy rate, clinical pregnancy rate, embryo implantation rate, and early abortion rate among the severe vitamin D deficiency, vitamin D deficiency, and non-vitamin D deficiency groups for patients with normal ovarian reserve. Ongoing debate surrounds the function of vitamin D in ART. It is necessary to conduct further studies exploring the relationships between vitamin D levels and pregnancy outcomes in a larger patient population and identify patients who would truly benefit from vitamin D supplementation.

## Data availability statement

The original contributions presented in the study are included in the article/**Supplementary Material**. Further inquiries can be directed to the corresponding author.

## Ethics statement

The studies involving humans were approved by the institutional review board of the Affiliated Taizhou People’s Hospital to Nanjing Medical University. The studies were conducted in accordance with the local legislation and institutional requirements. Written informed consent for participation was not required from the participants or the participants’ legal guardians/next of kin in accordance with the national legislation and institutional requirements.

## Author contributions

Study design: HG, JW, and RL. Data collection: YY, CX, MY, and RL. Data analysis and interpretation: RL and JHW. Original manuscript writing: RL, YY, and JHW. Manuscript content review: MY, DZ, PZ, JW, and HG. Final approval and accountable for all aspects of the work: All authors. All authors contributed to the article and approved the submitted version.
