# Supplementary material for: The role of serum vitamin D in patients with normal ovarian reserve undergoing the first IVF/ICSI cycle
Source: Front Endocrinol (Lausanne). 2023 Aug 24;14:1249445. doi: 10.3389/fendo.2023.1249445 (PMC10491894; doi:10.3389/fendo.2023.1249445)
Supplement: Supplementary file 1 [file Table_1.docx]

Supplementary Material

The role of serum vitamin D in patients with normal ovarian reserve undergoing the first IVF/ICSI cycl

Rong Luo, Jiahui Wang, Yu Yang, Cen Xu, Minyan Yang, Dandan Zhu, Jia Wang, Ping Zhang, Hongshan Ge^*^

*** Correspondence:** Hongshan Ge: hongshange@njmu.edu

# Supplementary Tables

Supplementary Table 1. Univariable analysis of the primary outcome

| **Parameter** | **Clinical pregnancy (n = 141)** | **Non-clinical pregnancy (n = 123)** | ***P* Value** |
| --- | --- | --- | --- |
| Age (years) | 29 (27, 32) | 30 (27, 33) | 0.112 |
| Infertility duration (years) | 3 (2, 4) | 2 (1, 4) | 0.640 |
| Season of embryo transfer (%) |  |  |  |
| Winter | 29 (20.6) | 27 (22.0) | Ref |
| Spring | 50 (35.5) | 43 (35.0) | 0.870 |
| Summer | 32 (22.7) | 20 (16.3) | 0.803 |
| Autumn | 30 (21.3) | 33 (26.8) | 0.029 |
| 25OH-D group |  |  |  |
| 25OH-D < 10 ng/ml | 28 (19.9) | 26 (21.1) | Ref |
| 10 ng/ml ≤ 25OH-D < 20 ng/ml | 93 (66.0) | 79 (64.2) | 0.778 |
| 25OH-D ≥ 20 ng/ml | 20 (14.2) | 18 (14.6) | 0.941 |
| BMI (kg/m^2^) | 22.80 (20.38, 26.77) | 22.50 (20.60, 26.40) | 0.794 |
| Basal FSH (IU/L) | 6.14 (5.23, 7.58) | 6.54 (5.42, 7.97) | 0.196 |
| Basal LH (IU/L) | 4.62 (2.99, 6.31) | 4.31 (2.84, 6.23) | 0.818 |
| Basal E_2_ (pg/ml) | 30.89 (25.54, 42.60) | 31.11 (23.81, 45.16) | 0.607 |
| AMH (ng/ml) | 3.88 (2.97, 6.32) | 3.43 (2.16, 5.64) | 0.104 |
| AFC | 18 (14, 22) | 16 (12, 21) | 0.022 |
| Primary etiology |  |  |  |
| Tubal factor | 54 (38.3) | 53 (43.1) | Ref |
| Ovulatory dysfunction | 23 (16.3) | 20 (16.3) | 0.735 |
| Endometriosis | 10 (7.1) | 5 (4.1) | 0.177 |
| Uterine factor | 3 (2.1) | 3 (2.4) | 0.982 |
| Sperm factor | 19 (13.5) | 18 (14.6) | 0.926 |
| Unexplained fertility | 32 (22.7) | 24 (19.5) | 0.408 |
| Gravidity |  |  |  |
| 0 | 90 (63.8) | 69 (56.1) | Ref |
| 1 | 33 (23.4) | 24 (19.5) | 0.865 |
| ≥ 2 | 18 (12.8) | 30 (24.4) | 0.038 |
| Parity |  |  |  |
| 0 | 120 (85.1) | 96 (78.0) | Ref |
| 1 | 19 (13.5) | 25 (20.3) | 0.169 |
| ≥ 2 | 2 (1.4) | 2 (1.6) | 0.834 |
| Ovarian stimulation protocol |  |  |  |
| GnRH agonist | 76 (53.9) | 60 (48.8) | Ref |
| GnRH antagonist | 61 (43.3) | 54 (44.9) | 0.654 |
| PPOS | 4 (2.8) | 9 (7.3) | 0.158 |
| Fertilisation method |  |  |  |
| IVF | 113 (80.1) | 95 (77.2) | 0.575 |
| ICSI | 28 (19.9) | 28 (22.8) | Ref |
| No. of embryos transferred |  |  |  |
| 1 | 21 (14.9) | 29 (23.6) | Ref |
| 2 | 120 (85.1) | 94 (76.4) | 0.102 |
| Phase of embryo transferred (%) |  |  |  |
| Cleavage embryo | 53 (37.6) | 73 (59.3) | Ref |
| Blastocyst | 88 (62.4) | 50 (40.7) | 0.001 |
| Transfer type (%) |  |  | 0.481 |
| Fresh | 41 (29.1) | 31 (25.2) | 0.471 |
| Frozen | 100 (70.9) | 92 (74.8) | Ref |
| Endometrial thickness (mm) | 10.1 (9.0, 11.5) | 9.3 (8.5, 11.10) | 0.031 |

Note: 25OH-D, 25-hydroxyvitamin D; BMI, body mass index; FSH, follicle stimulating hormone; LH, luteinizing hormone; E_2_, estradiol; AFC, antral follicle count; AMH, anti-mullerian hormone; GnRH, gonadotrophin-releasing hormone; PPOS, progestin-primed ovarian stimulation; IVF, in vitro fertilization; ICSI, intracytoplasmic sperm injection; No., number.

Supplementary Table 2. Univariable linear regression of the number of 2PN

| **Parameter** | **Univariable linear regression Coefficient 95%CI** | ***P* Value** |
| --- | --- | --- |
| Age (years) | -0.219 (-0.362, -0.076) | 0.003 |
| Infertility duration (years) | -0.260 (-0.582, 0.062) | 0.114 |
| Season of blood sampling | -0.453 (-1.009, 0.104) | 0.110 |
| Winter |  |  |
| Spring |  |  |
| Summer |  |  |
| Autumn |  |  |
| 25OH-D group | -0.569 (-1.607, 0.469) | 0.282 |
| 25OH-D < 10 ng/ml |  |  |
| 10 ng/ml ≤ 25OH-D < 20 ng/ml |  |  |
| 25OH-D ≥ 20 ng/ml |  |  |
| BMI (kg/m^2^) | -0.049 (-0.180, 0.082) | 0.462 |
| Basal FSH (IU/L) | -0.208 (-0.467, 0.051) | 0.115 |
| Basal LH (IU/L) | 0.104 (-0.038, 0.246) | 0.152 |
| Basal E_2_ (pg/ml) | 0.014 (-0.013, 0.041) | 0.320 |
| AMH (ng/ml) | 0.678 (0.456, 0.899) | 0.000 |
| AFC | 0.259 (0.171, 0.347) | 0.000 |
| Gravidity | -0.978 (-1.753, -0.203) | 0.014 |
| 0 |  |  |
| 1 |  |  |
| ≥ 2 |  |  |
| Parity | -1.185 (-2.585, 0.216) | 0.097 |
| 0 |  |  |
| 1 |  |  |
| ≥ 2 |  |  |
| Ovarian stimulation protocol | 0.208 (-0.829, 1.245) | 0.693 |
| GnRH agonist |  |  |
| GnRH antagonist |  |  |
| PPOS |  |  |
| Fertilization method | -0.946 (-2.437, 0.544) | 0.212 |
| IVF |  |  |
| ICSI |  |  |

Note: 25OH-D, 25-hydroxyvitamin D; BMI, body mass index; FSH, follicle stimulating hormone; LH, luteinizing hormone; E_2_, estradiol; AFC, antral follicle count; AMH, anti-mullerian hormone; GnRH, gonadotrophin-releasing hormone; PPOS, progestin-primed ovarian stimulation; IVF, in vitro fertilization; ICSI, intracytoplasmic sperm injection.
